# Biology and physiology of Calbindin-D9k in female reproductive tissues: Involvement of steroids and endocrine disruptors

**DOI:** 10.1186/1477-7827-3-66

**Published:** 2005-11-16

**Authors:** Kyung-Chul Choi, Peter CK Leung, Eui-Bae Jeung

**Affiliations:** 1 Department of Obstetrics and Gynecology, British Columbia Children's and Women's Hospital, Child and Family Research Institute, University of British Columbia, Vancouver, BC V6H 3V5 Canada; 2 Laboratory of Veterinary Biochemistry and Molecular Biology, College of Veterinary Medicine and Research Institute of Veterinary Medicine, Chungbuk National University, Cheongju, Chungbuk, 361-763 Republic of Korea

## Abstract

Although Calbindin-D9k (CaBP-9k), a cytosolic calcium binding protein which has calcium binding sites, is expressed in various tissues, i.e., intestine, uterus, and placenta, potential roles of this gene and its protein are not clearly understood. Uterine CaBP-9k may be involved in controlling myometrial activity related with intracellular calcium level and is not under the control of vitamin D despite the presence of vitamin D receptors. But, it is under the control of the sex steroid hormones, estrogen (E2) and progesterone (P4), in female reproductive systems including the uterus and placenta. Thus, in this review, we summarize recent research literature in regards to the expression and regulation of CaBP-9k in mammals and introduce the research data of recent studies by us and others.

## Introduction

A 9-kilodalton cytosolic calcium-binding protein termed as Calbindin-D9k (CaBP-9k) belongs to a family of intracellular proteins which have high affinities for calcium, and has two calcium binding domains [1]. The full-length cDNA encoding the human CaBP-9k has been cloned using reverse transcription/PCR, which includes coding region of 79 amino acids, 57 nucleotides 5'- and 159 nucleotides 3'-non-coding region, and a poly(A) tail (total 600 nucleotides in length) [2]. Further, our study revealed that this gene spans about 5.5-kb and is localized on the X-chromosome, consists of three exons and carries four Alu repeats [3]. In addition to its genomic structure, a sequence of 50 nucleotides downstream from the promoter showed an extensive homology to the estrogen response element (ERE) at the same location within the rat calbindin-D9k gene, suggesting that a two-nucleotide change within this region in human causes the gene to lack expression in human uterus and placenta [3].

It has been demonstrated that CaBP-9k is expressed in diverse mammalian tissues, i.e., intestine, uterus, kidney, and bone [4-7]. The functional role of CaBP-9k is involved in intestinal calcium absorption and its gene is regulated at the transcriptional or post-transcriptional level by 1,25-dihydroxyvitamin D3 (1,25-(OH)_2_D_3_), a hormonal form of vitamin D [8,9]. This hormonal form caused a parallel increase in CaBP-9k mRNA and intestinal absorption of calcium in rats [10]. In addition, uterine CaBP-9k may be involved in controlling myometrial activity related with intracellular calcium level [6], but an exact role of CaBP-9k in the uterus is still under investigation by us and a few of other research groups. Recently, we demonstrated that uterine CaBP-9k is responsive to exogenous estrogen (E2) and can be a biomarker for environmental estrogenic chemicals, so called as "endocrine disruptors" in rat models [11-15]. Thus, in this review, we summarize recent research literature in regards to the expression and regulation of CaBP-9k in mammals and introduce updated research results by us and others.

## Uterine expression of CaBP-9k

It has been demonstrated that CaBP-9k is mainly expressed in the endometrial stroma and myometrium of the uterus in non-pregnant rats [16,17], whereas this gene is translocated into the epithelium of the uterus in pregnant rats [18]. However, it has been shown that the CaBP-9k is only expressed in the luminal and glandular epithelium of the endometrium, not in the myometrium and in the stromal cells of the endometrium in non-pregnant cows [19]. In contrast to the regulation of CaBP-9k in the intestine, CaBP-9k gene is not under the control of vitamin D in the uterus despite the presence of vitamin D receptors in this tissue. This gene appears to be under the control of the sex steroid hormones [17,20,21].

**Figure 1:**
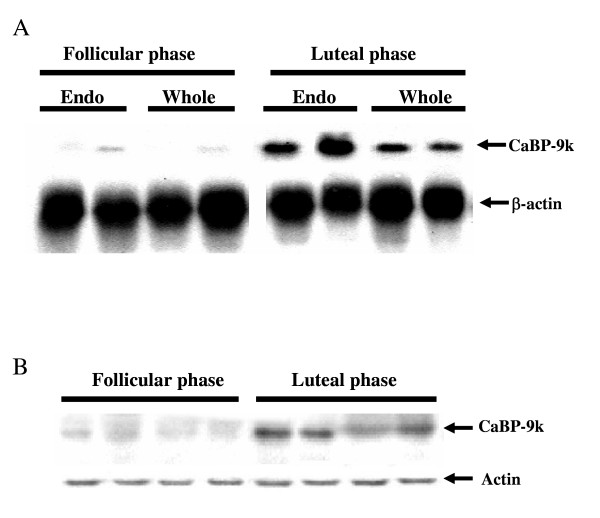
Porcine CaBP-9k mRNA expression in the endometrium and whole uterus during an estrus cycle. To investigate a role of CaBP-9k in the tissue compartment of the pig uterus, the expression levels of CaBP-9k mRNA (**A**) and protein (**B**) were analyzed by Northern blot and immunoblot analyses. [Reproduced with permission from Yun S-M, Choi KC, Kim IH, An BS, Lee GS, Hong EJ, Son JH, Oh GT, Jeung E-B 2004 Calbindin-D_9k _mRNA expression and regulation during estrus cycle in the pig uterus. *Mol Reprod Dev *67: 251\endash 256]

There is a strong body of evidence that CaBP-9k is regulated by sex steroid hormones in the uterus of rats. Treatment of 21-day-old rats with E2 resulted in an increase in the expression of CaBP-9k mRNA up to 300-fold and its mRNA was shown to fluctuate in the uterus of rats during estrous cycle, where serum E2 level was also under fluctuation [22]. Although the expression of CaBP-9k mRNA is not detectable at diestrus when E2 level is at the lowest, this mRNA increases at proestrus and reaches the highest level at estrus in response to the rise in plasma E2 and then decreases at metestrus in the uterus of rats [20,22]. In addition, E2-dependent regulation of CaBP-9k gene was demonstrated. i.e., CaBP-9k synthesis decreased drastically in the uterus of ovariectomized rats, whereas it was greatly enhanced by low physiological doses of E2 in a dose-dependent manner by CaBP-radioimmunoassay (RIA) [17]. This E2-dependent regulation of CaBP-9k gene was also approved in the uterus of mature ovariectomized and immature rats by slot and Northern blot analysis [23,24]. In estrogen-primed ovariectomized rats, progesterone (P4) inhibited E2-induced CaBP-9k gene expression, which was completely abolished by co-administration of RU486, a P4 antagonist [20]. In the pregnant rats, P4 was shown to be responsible for down-regulation of CaBP-9k gene in the uterus during early pregnancy [25]. In the ovariectomized (OVX) gilts, E2 treatment induced an increase in CaBP-9k mRNA level, whereas P4 administration to ovariectomized pigs decreased CaBP-9k mRNA levels [26]. Recently, we demonstrated that CaBP-9k mRNA and protein are dominantly expressed during luteal phase, indicating that P4 may play an important role in the up-regulation of CaBP-9k gene in the porcine uterus during luteal phase, which is unlike the condition in the rat uterus (Fig. 1) [27]. In this study, the porcine CaBP-9k may be dominantly expressed in the epithelium and glandular structure of the porcine uterus during luteal phase, suggesting that CaBP-9k gene may also be differentially regulated during this cycle presumably by steroid hormones, especially up-regulated P4 level in this tissue [27]. In addition, a positive role of P4 on the expression of CaBP-9k has been demonstrated in the bovine uterus, which indicates that the expression of CaBP-9k was greatest during P4-dominated luteal phase of the bovine estrous cycle [19].

In contrast to the uterus of rats, the expression of CaBP-9k gene is not under strict E2 regulation in the uterus of mice. The uterus of mice has been demonstrated to express CaBP-9k and its level increases in this tissue during early pregnancy and implantation [28,29]. CaBP-9k mRNA is expressed in the endometrial epithelia, both luminal and glandular, in the uterus at the time of implantation, and in the luminal, but not in the glandular, epithelia on early pregnancy (day 5 of pregnancy). P4 enhanced CaBP-9k mRNA expression in the uterus, whereas E2 did not in the oophorectomized adult mice [28]. A higher expression of CaBP-9k mRNA was observed in the uterus of mice at diestrus and metestrus, whereas only basal level of its expression at proestrus and estrus, and E2 alone did not induce uterine CaBP-9k mRNA in this study [29]. Taken together, these results suggest the complex hormonal regulation of CaBP-9k in the uterus of different species. To date, there was no evidence that CaBP-9k gene may be regulated by E2 in female reproductive tract of mice. In the recent study, we demonstrated that RU486, a P4 antagonist, induced a significant decrease in CaBP-9k mRNA expression, whereas tamoxifen and ICI 182,780, an E2 antagonists, had no effect on CaBP-9k mRNA expression, suggesting that P4, not E2, is a key regulator of CaBP-9k mRNA expression during late pregnancy and lactation in the uterus of mice [30].

The mechanism involved in the regulation of uterine CaBP-9k gene by steroids is relatively well understood in rats. In the uterus of rats, estrogen is known to up-regulate and progesterone down-regulate the expression of CaBP-9k gene during estrous cycle and early pregnancy [20,22,24,25]. The expression level of CaBP-9k mRNA fluctuates during the estrous cycle, but shows very different expression pattern in the uterus of between rats and mice as abovementioned [29]. The mechanism of distinct regulation of CaBP-9k gene between the rat and mouse is not clear at the moment. The effect of E2 on the regulation of CaBP-9k appears to be mediated through an imperfect estrogen-responsive-like element (ERE) identified in the intron A of mouse CaBP-9k gene [8,24]. The regulation of this gene is known to be mediated by an E2 response element located at the first intron of rat CaBP-9k gene [31]. Recently, cloning of intron A of the mouse CaBP-9k gene have revealed single-base difference in the ERE compared to that of the rat [32]. This may partially explain the observed difference in the hormonal regulation of CaBP-9k gene in the uterus of between rats and mice. In addition, a distinct regulation of porcine CaBP-9k gene in the uterus is explained by no presence of a functional ERE within intron A region [26]. However, we can not rule out the possibility of involvement of other unknown cell-, tissue-, and species-specific factors in the CaBP-9k gene expression. This idea is supported by the finding that E2 regulation of CaBP-9k gene in rats was only uterine-specific and this does not occur in the intestine [17]. Although the putative ERE failed to bind to the estrogen receptor (ER) from the mouse uterus, we isolated mouse genomic clones of the CaBP-9k gene and analyzed their expression in the mouse uterus [33]. In addition, we found a promoter region of CaBP-9k gene containing the putative progesterone response element (PRE) and its expression was stimulated by P4, suggesting that the mouse uterine CaBP-9k gene is expressed under the control of a PRE [33]. In the recent study, we demonstrated that P4 and PR may be a dominant factor in the regulation of CaBP-9k and E2 and ERα can also influence the expression of CaBP-9k gene via an indirect pathway in the uterus of immature mice [34]. Currently, endocrine disruptor-induced expression of CaBP-9k mRNA and protein was reversed or abolished by pretreatment with RU486 or ICI 182,780, suggesting that these synthetic chemicals may have both progestogenic and estrogenic properties by acting through PR or ER in the induction of uterine CaBP-9k mRNA and protein in the uterus of immature mice [35].

## Uterine induction of CaBP-9k as a biomarker for endocrine disruption

Endocrine disruptors (EDs) are environmental chemicals that interfere with physiological systems, adversely affecting hormone balance (endocrine system) or disrupting normal function in the organs that hormones regulate or modulate, for example, female and male reproductive system [36]. Representative example of suspected environmental estrogenic EDs includes the drugs which have been specifically designed to treat hormone imbalance in humans. These estrogenic compounds, including octylphenol (OP), nonylphenol (NP), bisphenol A (BPA), and methoxychlor (MXC), can also be transferred through the placenta to the fetus and through breast milk to infants [11,13,37]. Screening methods to detect endocrine disrupters have been evaluated by many groups, i.e. the receptor binding assay, reporter gene assay, and immature rat uterotrophic assay. The reporter gene assay has many benefits as a promising prescreening procedure, because this assay could be performed as a high throughput screening process to detect an endocrine disruptor from thousands of chemicals and no use of experimental animals is required [38]. To screen estrogenic chemicals in the induction of endocrine disruption, genetically sensitive animal models, mice and rats, should be developed. Efforts to identify the mechanisms of endocrine disruption by estrogenic chemicals need to be supported for optimal test methods for thousands of potential chemicals in reproductive development and function [39]. Thus, we have recently established a sensitive method to detect CaBP-9k mRNA and protein using immature rats, which can be used as a biomarker for endocrine disruptors, thus, we introduce our current results in regard to estrogenic effect of endocrine disruptors in the uterus of immature rats [12]. Among the assays for the estrogenic activity of chemicals, an assay to detect an endogenous gene expression that measures estrogen-induced changes either in cultured cells or in selected tissues from exposed animals has been proposed and is widely being used. In our previous study, we demonstrated that phthalate esters exhibit a weak estrogenic activity *in vitro *assay at high concentrations. Although phthalates resulted in an increase in MCF-7 cell proliferation by estrogenic effect, they could not induce CaBP-9k expression *in vivo *system following oral treatments, assuming that these phthalates are easily metabolized to inactive forms *in vivo *system. These results suggest that a conflict may exist in estrogenic effect by various phthalates between *in vitro *and *in vivo *models related to the expression of CaBP-9k [40].

**Figure 2:**
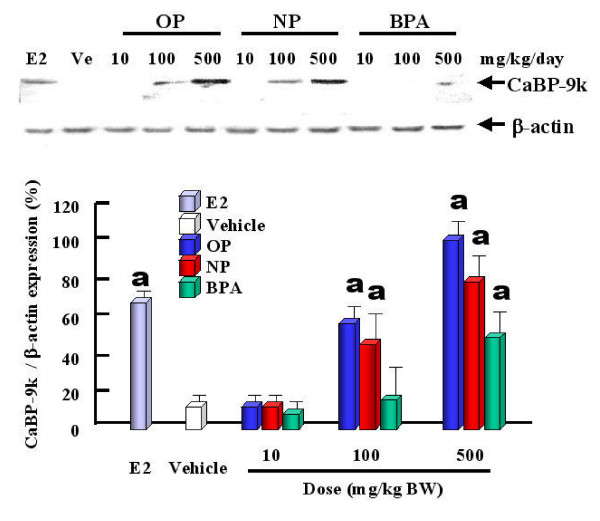
Induction of uterine CaBP-9k protein by estrogenic compounds, i.e., OP, NP and BPA was assessed in a dose-dependent manner at 24 h after final injection by immunoblot analysis. Data are presented as the mean ± SD. a; significantly different compared with vehicle at P < 0.05. [Reproduced with permission from An B-S, Choi KC, Kang SK, Hwang WS, Jeung EB 2003 Novel Calbindin-D9k protein as a useful biomarker for environmental estrogenic compounds in the uterus of immature rats. *Reprod Toxicol *17: 311–319]

**Figure 3:**
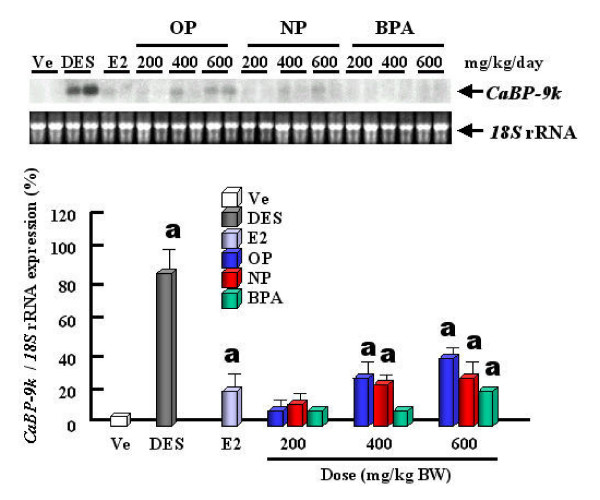
Induction of CaBP-9k mRNA expression in fetal uterus by estrogenic compounds. The values represent means ± SD. a, P < 0.05 vs. vehicle. [Reproduced with permission from Hong EJ, Choi KC, Jeung E-B 2003 Maternal-fetal transfer of endocrine disruptors in the induction of Calbindin-D9k mRNA and protein during pregnancy in rat model. *Mol Cell Endocrinol *212: 63–72]

**Figure 4:**
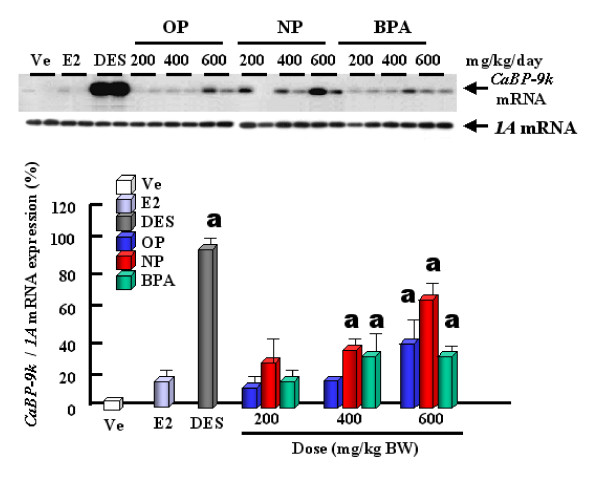
Effect of endocrine disruptors on the induction of CaBP-9k mRNA in neonatal uterus. RT-PCR/Southern blot analysis for CaBP-9k mRNA on day 6 of lactation was performed as previously described. The values represent means ± SD (n = 5). a, P < 0.05 vs. vehicle (VE). [Reproduced with permission from Hong E-J, Choi KC, Jung YW, Leung PCK, Jeung E-B 2004 Transfer of maternally injected endocrine disruptors through breast milk during lactation induces neonatal Calbindin-D9k in the rat model. *Reprod Toxicol *18: 661–668]

**Figure 5:**
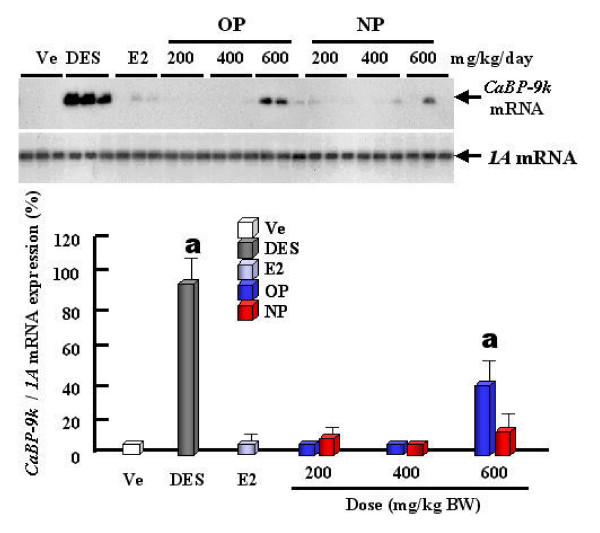
Effect of OP and NP on the induction of CaBP-9k mRNA in neonatal uterus. RT-PCR/Southern blot assay was performed during lactation period in neonatal uterus. The values represent means ± SD. a, P < 0.05 vs. vehicle. [Reproduced with permission from Hong EJ, Choi KC, Jeung EB 2004 Induction of Calbindin-D_9k _mRNA and protein by maternal exposure to alkylphenols during late pregnancy in maternal and postnatal uteri of rats. *Biol Reprod *71: 669–675]

**Figure 6:**
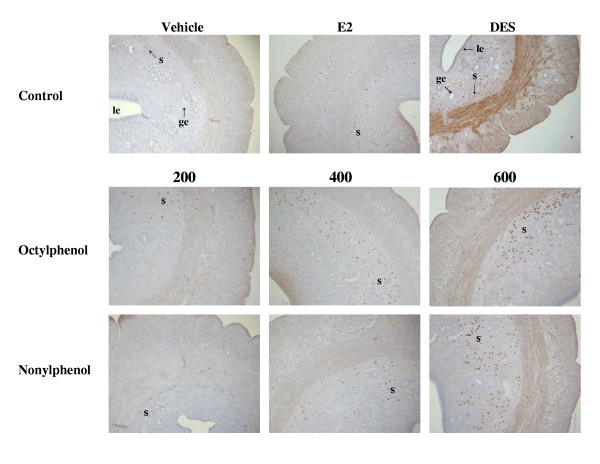
Localization of CaBP-9k protein by immunohistochemicalstaining in maternal uterus. Immuno-reactivity of CaBP-9k protein expression following treatment with OP and NP was investigated in endometrium and smooth myometrial fibers dose-dependently. Especially, these proteins that are widely spaced through the stromal cells in endometrium s, stroma cells; le, Luminal epithelial cell; ge, glandular epithelial cell. Magnification × 100. [Reproduced with permission from Hong EJ, Choi KC, Jeung EB 2004 Induction of Calbindin-D_9k _mRNA and protein by maternal exposure to alkylphenols during late pregnancy in maternal and postnatal uteri of rats. *Biol Reprod *71: 669–675]

The expression levels of CaBP-9k mRNA and protein are strongly up-regulated by estrogenic compounds (OP, NP and BPA) and E2 itself in the uterus of immature rats (Fig. 2), indicating that CaBP-9k can be a useful biomarker for detection of the estrogenicity of putative estrogenic compounds. Thus, regarding to risk assessment, we propose that CaBP-9k mRNA and protein assay in the immature rat uterus can be a very sensitive and powerful tool to identify compounds with estrogenic activity when used in combination with classical assays [11,23]. Treatments of dams with OP, NP and BPA resulted in an increase of CaBP-9k mRNA and protein in maternal and fetal uteri (Fig. 3) of immature rats [13]. These results demonstrate that maternally injected estrogenic compounds resulted in an increase of CaBP-9k mRNA and/or protein in the maternal tissues (uterus and placenta) and fetal uterus during late pregnancy, suggesting that placenta may not be a reliable barrier against these estrogenic compounds for fetal health [13]. The uterus is a highly estrogen-responsive tissue, which can be measured through changes in CaBP-9k expression. In addition, we investigated the potential for estrogenic compounds, OP, NP, BPA, diethylstilbestrol (DES) and E2 to be transferred through breast milk from the dam to the neonate during lactation via measuring the induction of CaBP-9k in uterine tissue [14]. These results indicate that these compounds have an estrogenic effect on the maternal uterus during the lactation period, as shown by the induction of both CaBP-9k mRNA and protein. There was a significant increase in CaBP-9k mRNA in neonatal uterus when the dams were treated with high doses of estrogenic compounds, but protein levels of CaBP-9k were undetectable (Fig. 4). Taken together, these findings suggest that maternally injected estrogenic compounds may be transferred to neonates through breast milk and thus, affect uterine function, as shown by the induction of CaBP-9k gene expression in neonatal uterus [14]. In addition, we recently examined the effect of OP, NP and BPA on the expression of CaBP-9k following maternal exposures during late pregnancy in maternal and fetal uterus [15,41]. The expression of CaBP-9k mRNA was also induced following treatment with a high dose (600 mg/kg BW) of OP transferred from mother exposed to fetuses during late pregnancy and persisted in 5 day of lactation (Fig. 5). In parallel with mRNA level, the expression level of CaBP-9k protein was significantly induced by treatment with a high dose of OP and NP in the maternal uterus by immunohistochemistry (Fig. 6). In conclusion, the maternal exposures to OP, NP and BPA during late pregnancy increased the expression levels of CaBP-9k mRNA and protein in maternal or neonatal uteri, suggesting that the absorption and distribution of environmental estrogenic compounds in maternal and neonatal uteri are extremely rapid, and these chemicals can easily pass though placenta during pregnancy to affect functions of neonatal reproductive tissues [15,41]. In addition, a novel in vivo model was introduced to detect both estrogenic and progestogenic activities of EDs in the induction of CaBP-9k mRNA and protein in the uterus of immature mice [35].

**Figure 7:**
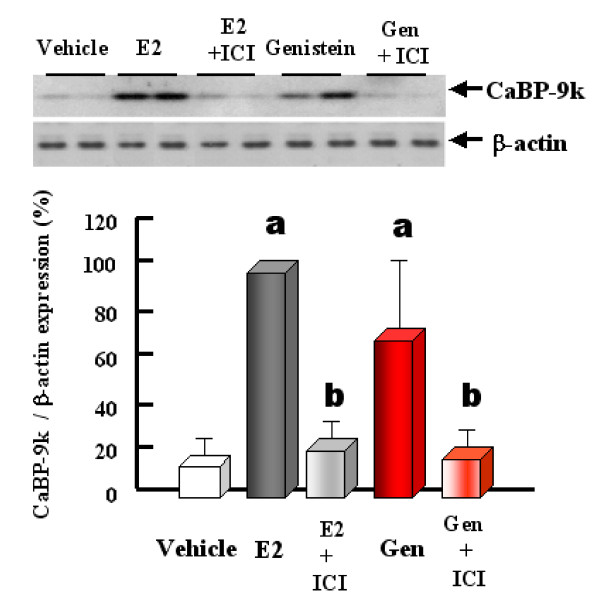
Effect of ICI on genistein-induced uterine CaBP-9k protein expression. Immature rats were injected SC with ICI at 30 min prior to genistein (40 mg/kg BW per day) or E2, and euthanized 24 h after final injection. The level of CaBP-9k protein was analyzed by immunoblot analysis. The values represent means ± SD. a, P < 0.05 vs. vehicle; b, P < 0.05 vs. genistein or E2 treatment only [Reproduced with permission from Lee GS, Choi KC, Kim HJ, Jeung EB 2004 Effect of genistein on the expression of Calbindin-D_9k _as a potential estrogenic compound in the uterus of immature rats through estrogen receptors. *Toxicol Sci *82: 451–457]

Genistein, a phytoestrogen, has been shown to have relatively 20-fold higher binding affinity to the ERβ than ER by a solid-phase binding assay [42]. To determine which ER is involved in the induction of *CaBP-9k *gene, we employed genistein as a potent ERβ agonist to clarify its effect on uterine CaBP-9k regulation [43]. Both CaBP-9k mRNA and protein levels were significantly induced by genistein in the uterus of immature rats. It is of interest that the pre-treatment of immature rats with ICI 182,780 (ICI), followed by genistein or E2, completely blocked genistein- and E2-induced CaBP-9k protein in this tissue as demonstrated in Fig. 7. In addition, genistein was demonstrated to induce ERα protein, but not ERβ or PR mRNA, an E2-responsive gene, in this tissue. These results imply that genistein, an ERβ ligand, may regulate *CaBP-9k *gene through ERα pathway and ERα may be a key mediator in the induction of uterine *CaBP-9k *gene in immature rats [43]. To support an involvement of ERα-dependent pathway by EDs, we demonstrated that uterine *CaBP-9k *gene expression is mainly mediated by propyl pyrazole triol (PPT), an ERα-selective ligand, in a dose- and time-dependent manner, in the uterus of immature rats [44]. In contrast, no significant alteration in the uterine *CaBP-9k *gene was observed after diarylpropionitrile (DPN), an ERβ-selective ligand. In addition, an estrogenicity of PPT in inducing *CaBP-9k *expression was completely blocked by ICI 182,780; which suggests that uterine *CaBP-9k *is solely enhanced though ERα. Taken together, these results indicate that uterine *CaBP-9k *is induced by E2 and endocrine disrupting chemicals via ERα pathway, but not ERβ, in the uterus of immature rats [44].

## Placental expression of CaBP-9k

Transport of Ca2+ through placenta is responsible for developing fetus, and CaBP-9k appears to play an important role in the regulation of Ca2+ from the mother to the fetus during pregnancy. However, the role of CaBP-9k is unknown to date in placenta during pregnancy. A recent study demonstrated that CaBP-9k transcript is present in cytotrophoblast cells and syncytiotrophoblasts of human term placenta, with a lower expression in cytotrophoblast cells as compared to syncytiotrophoblasts [45]. In addition, CaBP-9k protein was present in cytotrophoblast and syncytiotrophoblast placental tissue sections as well as in cultured cells, indicating that CaBP-9k is unequivocally expressed by trophoblast cells from human term placenta [45]. The expression of CaBP-9k gene has been investigated in the placenta of other species [26,46-48]. The high level of CaBP-9k has been localized to epithelial cells of the yolk sac and endodermal cells of the placenta [16]. The expression of CaBP-9k mRNA was not detectable by Northern blot analysis, while this transcript was detected in porcine myometrium and placenta by RT-PCR [26]. As previously described, CaBP-9k mRNA has been also localized in the trophoblasts in various species. It is hypothesized that CaBP-9k plays a role in calcium transfer and fetal growth by parallel gestational changes in placental CaBP-9k which reflects the fetal accumulation of calcium. An increased level of CaBP-9k gene in the caruncular epithelium during the last trimester is in response to the increasing need for calcium to supply the fetal skeleton with mineralization, suggesting that CaBP9k may play a role in transporting calcium across the placenta in cattle [48]. In the placenta of mice, the distinct regulation of CaBP-9k has been demonstrated in the placenta compared to other tissues such as intestine and kidney, indicating that the expression of this gene is not dependent on Vitamin D receptor [49]. Recently we demonstrated the expression of CaBP-9k for the first time in mouse placenta and extra-embryonic membrane separately, and CaBP-9k mRNA may be regulated by sex steroid hormones (E2 and P4) and their receptors through complex pathway in these tissues [50].

## Concluding Remarks

Although CaBP-9k is mainly expressed in female reproductive tissues, i.e., uterus and placenta of various species, the role of CaBP-9k remains unknown. It can be hypothesized that uterine CaBP-9k may be involved in controlling myometrial activity related with intracellular calcium level and placental CaBP-9k plays a role in calcium transfer from the mother to the fetus for fetal growth. It appears that CaBP-9k gene is not under the control of vitamin D in the uterus despite the presence of vitamin D receptors in this tissue; instead it is under the control of the sex steroid hormones. The hormonal mechanism controlling uterine CaBP-9k gene is relatively well understood in the rat. In the uterus of rats, estrogen is known to up-regulate and progesterone down-regulate the expression of CaBP-9k gene during estrous cycle and pregnancy. However, the recent studies demonstrated that CaBP-9k is mainly regulated by progesterone, not estrogen in the uterus of mice because of lack of responsiveness caused by a single-base difference in the ERE of mouse CaBP-9k gene compared to that of rats. Until now, a few studies demonstrated the expression and regulation of CaBP-9k gene in the placenta of various species. It appears that CaBP-9k mRNA may be regulated by sex steroid hormones (E2 and P4) and their receptors through complex pathway in these tissues. We assumed that ERα may be a key mediator in uterine *CaBP-9k *gene induction in immature rats. The elucidation of other factors that regulate CaBP-9k mRNA will further provide insight into the understanding of regulation of CaBP-9k in these tissues, and its roles in the control of reproductive functions.

The uterus is a highly estrogen-responsive tissue, which can be measured through changes in CaBP-9k expression. We demonstrated that the expression levels of CaBP-9k mRNA and protein are induced by estrogenic chemicals, so called "endocrine disruptors", in the uterus of immature rats. In addition, maternally injected estrogenic compounds resulted in an increase of CaBP-9k mRNA and/or protein in the fetal uterus during late pregnancy, suggesting that placenta may not be a reliable barrier against these estrogenic compounds for fetal health. It is of interest that maternally injected estrogenic compounds may be transferred to neonates through breast milk and thus affect uterine function, as shown by the induction of CaBP-9k gene expression in neonatal uterus. The expression of CaBP-9k mRNA and/or protein is an excellent biomarker to detect an estrogenic chemical in the uterus of immature rats which we developed and established. Availability of this gene using immature rats will provide an insight of risk assessment for estrogenic and progestogenic chemicals in our environment.
